# Ein Modell für Pretreatment Assessment und Prähabilitation bei Kopf-Hals-Tumoren: Vorstellung des Zürcher Prähabilitationskonzepts

**DOI:** 10.1007/s00106-026-01760-2

**Published:** 2026-04-22

**Authors:** Miriam E. F. van Beek, U. Colotto, M. Brockmann-Bauser, C. D. Thüring, S. A. Müller, G. B. Morand, J. E. Bohlender

**Affiliations:** 1 Klinik für Ohren‑, Nasen‑, Hals- und Gesichtschirurgie, Abt. Phoniatrie und Klinische Logopädie, Universitätsspital Zürich, Frauenklinikstrasse 24, 8091 Zürich, Schweiz; 2 Klinik für Ohren‑, Nasen‑, Hals- und Gesichtschirurgie, Universitätsspital Zürich, Zürich, Schweiz; 3 Universität Zürich, Zürich, Schweiz; 4 Lehrstuhl für Logopädie, Pädagogische Hochschule Weingarten, University of Education, Weingarten, Deutschland

**Keywords:** Dysphagie, Lebensqualität, Radiotherapie, Logopädie, Interdisziplinäre Kommunikation, Deglutition disorders, Quality of life, Radiotherapy, Speech language pathology, Interdisciplinary communication

## Abstract

**Hintergrund:**

Die Behandlung von Patienten mit Kopf-Hals-Tumoren, insbesondere im Zusammenhang mit Radio(chemo)therapie (R[C]T), führt häufig zu Einschränkungen der Schluckfunktion, des Ernährungszustands und der Lebensqualität. Am Universitätsspital Zürich (USZ) wurde eine logopädisch-phoniatrische Sprechstunde zur prätherapeutischen Untersuchung und Behandlung eingeführt, um (Spät‑)Folgen der Tumorbehandlung potenziell vorzubeugen oder zu reduzieren. Obwohl das Modell ursprünglich nicht auf die Übertragbarkeit auf andere Einrichtungen ausgerichtet war, könnte es als Beispiel für ähnliche Konzepte in anderen Kliniken dienen.

**Zielsetzung:**

Im vorliegenden Artikel wird ein strukturiertes Sprechstundenmodell zur Betreuung vor und nach einer R(C)T oder Operation vorgestellt. Der Fokus liegt auf praktischen Aspekten wie der Ressourcenplanung, Patientenzahlen und Herausforderungen bei der Umsetzung.

**Methoden:**

Das Modell wurde auf Basis einer Literaturrecherche und praktischer Erfahrungen entwickelt. Es definiert Zeitpunkte für Erstuntersuchungen, Nachkontrollen und therapeutische Interventionen. Seit 2019 ist es Bestandteil des interdisziplinären Kopf-Hals-Tumorzentrums am USZ.

**Ergebnisse:**

Seit 2019 wurden mehr als 400 Patienten betreut. Eine beispielhafte Analyse der Daten von 2024 zeigt, dass etwa 70 % der Patienten während der R(C)T die logopädischen Termine wahrnahmen. Drei Monate nach der R(C)T betrug die Teilnahme an den Kontrollen 76 %, nach einer Operation 94 %. Diese hohe Wahrnehmungsquote spiegelt die Akzeptanz des Modells wider. Herausforderungen bei der Integration und Durchführung betreffen v. a. die Ressourcenplanung und die interdisziplinäre Zusammenarbeit.

**Schlussfolgerung:**

Das vorgestellte Sprechstundenmodell bietet eine Struktur zur logopädischen Betreuung von Kopf-Hals-Tumorpatienten. Die erfolgreiche Umsetzung am USZ zeigt, dass es als Beispiel für ähnliche Konzepte in anderen Kliniken dienen kann. Die beschriebenen Herausforderungen und praktischen Überlegungen bieten Einsichten für die Implementierung solcher Modelle.

## Abstract (en)

**Background:**

The treatment of patients with head and neck tumors, particularly in the context of radio(chemo)therapy, R(C)T, often leads to impairments in swallowing function, nutritional status, and quality of life. At the University Hospital Zurich (USZ), a logopedic and phoniatric outpatient clinic was introduced for pretherapeutic assessment and treatment to potentially prevent or reduce the (late) effects of cancer treatment. Although the model was not initially designed for adaptation by other institutions, it could serve as an example for similar concepts in other clinics.

**Objective:**

This article presents an outpatient clinic model that provides structured logopedic care before and after R(C)T or surgery. The focus is on practical aspects, including resource planning, patient numbers, and challenges to implementation.

**Methods:**

The model was developed based on a literature review and practical experience. It defines timepoints for initial assessments, follow-up examinations, and therapeutic interventions. Since 2019, it has been part of the interdisciplinary head and neck cancer center at the USZ.

**Results:**

Since 2019, over 400 patients have been treated. A representative analysis of data from 2024 shows that approximately 70% of patients attended logopedic sessions during R(C)T. Three months after R(C)T, the attendance rate for follow-up assessments was 76%, and it was 94% for postoperative assessments. These high attendance rates reflect the model’s acceptance. Challenges related to integration and implementation primarily concern resource planning and interdisciplinary collaboration.

**Conclusion:**

The presented outpatient clinic model provides a structured approach to logopedic care for head and neck cancer patients. Its successful implementation at the USZ demonstrates that it can serve as an example for similar models in other clinics. The described challenges and practical considerations offer valuable insights for the implementation of such models in other settings.

Operationen und insbesondere Radio(chemo)therapien (R[C]T) zur Behandlung von Kopf-Hals-Tumoren (KHT) führen kurz- oder langfristig bei bis zu 70 % der Behandlungspopulation zu einer Dysphagie [15, 16, 25]. Diese kann auch Monate nach der Behandlung die Ernährung und sehr häufig die Lebensqualität stark beeinträchtigen [2, 15, 21]. Im Folgenden wird das Zürcher Prähabilitationskonzept vorgestellt: eine logopädisch-phoniatrische Sprechstunde mit Fokus auf die Dysphagie, deren Ziel es ist, Kurz- und Langzeitfolgen der Behandlung auf die Schluckfunktion zu vermeiden bzw. zu erkennen und zu verringern.

## Hintergrund

Unabhängig von der gewählten Therapiemethode (Operation mit/ohne adjuvante Radiotherapie, R[C]T) hat die Behandlung von Kopf-Hals-Tumoren (KHT) signifikante Auswirkungen auf die Schluckfunktion. Je nach Tumorlokalisation und Therapiemethode entwickeln bis zu 70 % der Patienten eine Dysphagie [15, 16, 26], die den Ernährungszustand, die Lebensqualität und die Mortalität erheblich beeinträchtigen kann [16, 28]. Akut treten während einer R(C)T Odynophagie, Xerostomie, Mukositis, Geschmacksverlust, Appetitlosigkeit und Nausea auf. Doch häufig persistieren selbst nach Beendigung der Therapie Xerostomie sowie andere orale und/oder pharyngeale Beschwerden. Diese Faktoren führen zu einer verringerten peroralen Nahrungsaufnahme und einem konsekutiven Muskelabbau durch die reduzierte Nutzung der Muskulatur. Erschwerend kommen (Spät‑)Folgen der Bestrahlung wie Lymphödem, Muskelatrophien, -fibrosen, Trismus und Sensibilitätsverlust hinzu [27].

Eine Dysphagie geht mit erheblichen psychosozialen Einschnitten einher

Eine Dysphagie geht zudem mit erheblichen psychosozialen Einschnitten einher. Durch die erschwerte Aufrechterhaltung der peroralen Nahrungsaufnahme unter einer R(C)T im Kopf-Hals-Bereich kann eine Abhängigkeit von Ernährungssonden während und nach der R(C)T entstehen. Eine besondere Herausforderung stellen hochgradige Dysphagien mehrere Jahre nach einer R(C)T für diese Patientengruppe dar, weshalb je nach Studie bis über 60 % der Patienten dauerhaft auf eine Sondenernährung angewiesen sind [18].

Bis zu 25 % der Betroffenen entwickeln zudem innerhalb von 5 Jahren nach R(C)T eine Aspirationspneumonie [15, 29]. Es handelt sich hierbei um erhebliche, teilweise vermeidbare bzw. beeinflussbare Risiken, die einer adäquaten Versorgung bedürfen.

Der entscheidende Zeitraum für optimale Rehabilitationsergebnisse könnte, gemäß verschiedenen Studien [1, 10, 13], bei Behandlung mittels (adjuvanter) R(C)T bereits vor dem Beginn der Behandlung liegen, wenn die Funktion noch weitgehend intakt ist. Ziel einer entsprechenden *Prä*habilitation ist es, Häufigkeit und Schweregrad akuter und zukünftiger Beeinträchtigungen als Folge der Therapie zu verringern, indem die orale Ernährung und die oropharyngealen Funktionen weitestgehend aufrecht erhalten [10] werden.

## Zürcher Prähabilitationskonzept

Im deutschsprachigen Raum fehlten hierfür lange überprüfbare Versorgungsmodelle. Aufgrund dessen wurde 2019 am Universitätsspital Zürich (USZ) das Zürcher Prähabilitationskonzept entwickelt [5–7] und 2020 in die interdisziplinäre Intake-Sprechstunde des Kopf-Hals-Tumorzentrums des USZ integriert. Es handelt sich dabei um eine logopädisch-phoniatrische Sprechstunde zur prätherapeutischen Untersuchung (Pretreatment Assessment), zur logopädischen Behandlung vor und während einer R(C)T (Prähabilitation) und zur Nachverfolgung (Posttreatment Assessment). Bis 2025 wurden im USZ bereits über 400 Patienten nach diesem Konzept untersucht und behandelt. Daher können die Erfahrungen und Herausforderungen anderen Kliniken bestenfalls bei der Etablierung ähnlicher Konzepte nützen und diese unterstützen.

In der Zwischenzeit zeigten sich auch anderweitig im deutschsprachigen Raum große Fortschritte in der Patientenversorgung bei KHT. So veröffentlichte 2024 die Gruppe um Kuenzel et al. ihr Protokoll für die PREHAPS-Studie [20] und erschien Mitte 2025 der CME-Beitrag „Prähabilitation“ und „enhanced recovery after surgery“ bei onkologischen Operationen im „Kopf-Hals-Bereich“ von Blaurock et al. [8]. Besonders hervorzuheben ist auch die im April 2025 veröffentlichte S3-Leitlinie „Oropharyngeale Dysphagie infolge einer Kopf-Hals-Tumor-Erkrankung“ [15] der Arbeitsgemeinschaft der Wissenschaftlichen Medizinischen Fachgesellschaften e. V. (AWMF).

### Ziele und Potenzial

Das Hauptziel der spezialisierten Sprechstunde ist es, die Häufigkeit und den Ausprägungsgrad von Dysphagien durch standardisierte Diagnostik, Beratung und Therapie bei Patienten mit KHT zu reduzieren.

Ein Pretreatment Assessment ermöglicht im Idealfall die Erfassung und das Management bereits bestehender aktueller subjektiver und/oder objektiver Schluckbeschwerden. Außerdem dient es der Prognose bezüglich der Schluck‑, Artikulations- und Stimmfunktion sowie einer gezielten logopädischen Therapie(planung) für Patienten vor R(C)T oder Operation.

Eine Prähabilitation ist eine proaktive Behandlung bei R(C)T und kann gemäß verschiedenen Studien insbesondere auf 3 zentrale Themenbereiche positive Auswirkungen haben:

1. *Bessere funktionelle Outcomes*
  durch u. a. eine verbesserte Funktion bzw. geringere Schädigungen schluckrelevanter Muskulatur sowie weniger Verschlechterungen von Schluckfunktion, Mundöffnung, Geschmackswahrnehmung und Speichelflussrate [10, 13, 14].
2. *Weniger Sondenabhängigkeiten *und schnellere Rückkehr zur oralen Nahrungsaufnahme [1, 13, 15, 17].
3. *Verbesserte Lebensqualität (QoL) und Patientenzufriedenheit in Bezug auf die Schluckfunktion* [1, 10, 12].

### Entwicklung

**Abb. 1:**
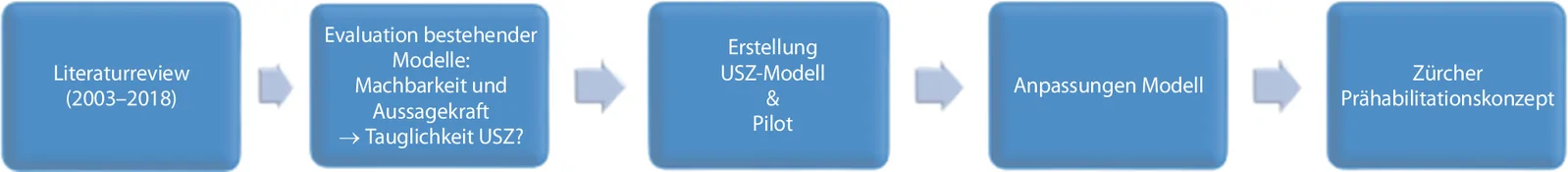
Entwicklung des Zürcher Prähabilitationskonzepts. *USZ* Universitätsspital Zürich

Basierend auf der Studienlage und den klinischen Erfahrungen der Abteilung Phoniatrie und Klinische Logopädie wurde 2019 das Zürcher Prähabilitationskonzept entwickelt und gestartet (Abb. 1).

Anhand einer Literaturrecherche über den Zeitraum 2003–2018 mit den Schlüsselwörtern „prehabilitation“, „pretreatment“, „proactive rehabilitation“, „dysphagia“, „swallowing“, „head and neck“, „HNC“, „radiation“, „chemoradiotherapy“ und „radiotherapy“ wurde evaluiert, welche Konzepte und/oder Behandlungsstrategien bereits beschrieben wurden und ob sie für die Umsetzung in unserer Sprechstunde geeignet sind. Basierend auf einer Auswertung von 24 Artikeln wurde ein USZ-spezifisches Modell erarbeitet, indem die Zeitpunkte für die Erstuntersuchung, Therapien und Kontrollen sowie Übungen für die betreffenden Tumorregionen definiert wurden.

Das erarbeitete Konzept wurde auf seine Praktikabilität im Klinikalltag getestet und fortlaufend an die Sprechstunde der Phoniatrie und Klinischen Logopädie der Otorhinolaryngologie (ORL) im USZ angepasst. Geeignete Patienten wurden initial durch die Logopädie im Tumorboard selektiert.

Das erarbeitete Konzept wurde auf seine Praktikabilität im Klinikalltag getestet

**Abb. 2:**
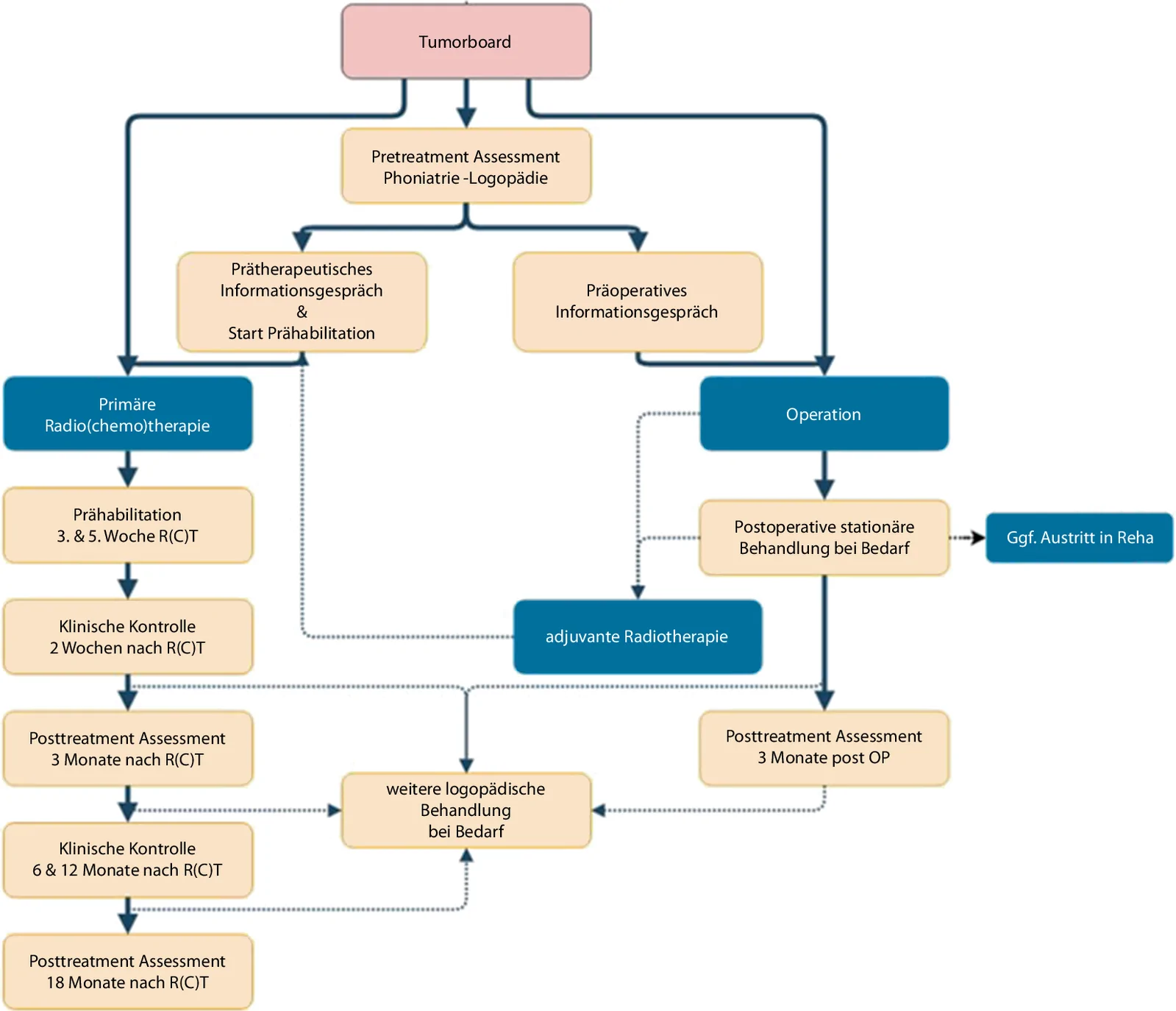
Ablauf des Zürcher Prähabilitationskonzepts (Adapt. nach [6])

Zeitgleich wurde im „Head & Neck-Team“ der ORL, Mund-Kiefer-Gesichtschirurgie (MKG) und Radioonkologie (RAO) des USZ die „interdisziplinäre Intake-Sprechstunde des Kopf-Hals-Tumorzentrums des USZ“ aufgebaut – eine Sprechstunde zur interdisziplinären prästationären Abklärung von Patienten mit Verdacht auf KHT [24]. In diesem Rahmen wurde unser Prähabilitationskonzept 2020 in diese Intake-Sprechstunde eingebettet. Zur weiteren Optimierung der Prozessabläufe erfolgten interne Fortbildungen in der ORL-Klinik. Nach letzten formalen Anpassungen der Zeitpunkte der Kontrolluntersuchungen (18 Monate statt 12 Monate nach Abschluss der R[C]T) läuft das Zürcher Prähabilitationskonzept seit August 2020 in seiner jetzigen Form (Abb. 2) ab.

Unabhängig von der Therapiewahl werden alle Patienten mit Erstkarzinomen in der Mundhöhle, im Oropharynx, Nasopharynx, Hypopharynx und/oder Larynx in unserer Sprechstunde untersucht und behandelt. Auch Patienten mit Rezidiven werden uns zugewiesen und gemäß diesem Konzept betreut.

### Ablauf und Inhalt

Nach klinischer Abklärung und Diagnosestellung eines KHT findet wöchentlich ein interdisziplinäres Tumorboard statt, in dem das weitere therapeutische Prozedere der betroffenen Patienten diskutiert und geplant wird.

Im Anschluss an das ärztliche Diagnosegespräch mit den Betroffenen erhalten alle neu zugewiesenen Patienten im Rahmen der Intake-Sprechstunde eine Beratung von Ernährungs- und Pflegeexperten sowie ein logopädisch-phoniatrisches Pretreatment Assessment. Die Untersuchung wird, wie auch in der S3-Leitlinie [15] empfohlen, nach einem standardisierten Ablauf inklusive FEES (flexible endoskopische Evaluation des Schluckakts) durchgeführt. Zur Auswertung verwenden wir das Protokoll Dynamic Imaging Grade of Swallowing Toxicity (DIGEST) [4]. Diese standardisierte Analysemethode stuft pharyngeale Dysphagien anhand der Parameter Sicherheit und Effizienz in Schweregrade ein und orientiert sich an dem universellen Rahmen für die Toxizitätsberichterstattung in onkologischen Studien (Common Terminology Criteria for Adverse Events, CTCAE).

Abhängig von der medizinischen Therapieempfehlung erfolgt hiernach ein präoperatives oder prätherapeutisches logopädisches Informationsgespräch und der Start der Prähabilitation.

Die Patienten werden danach entsprechend ihrem Behandlungsschema logopädisch betreut und kontrolliert.

#### Pretreatment Assessment

Das Pretreatment Assessment umfasst für alle Patienten:

- Anamnese mit Dokumentation von Gewicht und Ernährungssituation bzw. Koststufe
- Patient-Reported Outcome Measures (PROM) zur Schluckfunktion:
  - Deutsche Fassung des M.D. Anderson Dysphagia Inventory [3] (leitlinienkonform [15])
  - Sydney Swallowing Questionnaire (SSQ-G) [9] (leitlinienkonform [15])
  - Dysphagia Handicap Index [19]
- 100-ml-Wasserschlucktest (WST) [22, 23]
- Überprüfung und Dokumentation von Gesichtsmotorik, -sensibilität, Zungenkraft und -mobilität
- FEES (leitlinienkonform [15]) mit DIGEST-Protokoll [4] durch Logopädie

Zusätzlich:

1. Bei Tumoren in Mundhöhle, Oropharynx, Nasopharynx:
  - Kieferöffnungsmessung mittels TheraBite® Range of Motion Scale (Atos Medical, Malmö, Schweden)
  - Audioaufnahme der Artikulation (Reihen sprechen, Spontansprache)
2. Bei Hypopharynx- oder Larynxkarzinomen:
  - Standardisierte Stimmaufnahme (Sonagramm)
  - PROM zur Stimmfunktion
    – 9-Item Voice Handicap Index (VHI-9i) [11]
3. Bei Larynx- und Glottiskarzinomen findet neben der zuvor im Head & Neck Team erhobenen Diagnose eine ergänzende Beurteilung (Laryngostroboskopie) durch einen Phoniater statt.

#### Prätherapeutisches Informationsgespräch und Prähabilitationsmaßnahmen

Je nach gewählter Therapiemethode (Operation/R[C]T) werden die Patienten direkt im Anschluss an das Pretreatment Assessment leitlinienkonform [15] weiter betreut (Abb. 2).

##### Präoperatives Informationsgespräch.

Bei geplanter operativer Behandlung findet nach dem Pretreatment Assessment eine logopädisch fokussierte Aufklärung über die möglichen Folgen des operativen Eingriffs auf die Funktionen des Schluckens, Sprechens und der Stimme sowie das weitere logopädische Prozedere statt. Nach der Tumorresektion erfolgt eine stationäre Kontrolle und bei Bedarf stationäre und/oder ambulante logopädische Behandlungen (Abb. 2). Bei Bedarf werden bereits Schluckmanöver für die postoperative Phase instruiert.

##### Vorgehen bei R(C)T.

Bei geplanter R(C)T folgt direkt im Anschluss an das Pretreatment Assessment und somit circa 2 Wochen vor Bestrahlungsbeginn ein prätherapeutisches logopädisches Informationsgespräch, in dem Folgendes besprochen wird:

- Auswirkungen der R(C)T auf das Schlucken, Sprechen und/oder die Stimme
- Bedeutung der oralen Ernährung während der R(C)T für den Funktionserhalt gemäß den Prinzipien „use it or lose it“, „eat and exercise“ [17]
- Prophylaktische Empfehlungen zu ersten Maßnahmen bei Xerostomie und/oder Verschleimungsgefühl
- Ablauf der geplanten logopädischen Therapie- und Kontrolltermine

Zudem beginnt die Prähabilitation mit gezielten, standardisierten Übungen für die Muskulatur im Bereich der Tumorlokalisation ab diesem Zeitpunkt. Hierdurch sollen ein optimaler Muskelerhalt und eine schnellstmögliche bzw. weitestgehend normale Oralisierung und Schluckfunktion nach der Behandlung fazilitiert werden. Anhand der FEES des Pretreatment Assessments können zusätzlich individuelle Übungen und kompensatorische Maßnahmen erfolgen.

Der Zeitaufwand für das Pretreatment Assessment und die Prähabilitation bzw. das präoperative Informationsgespräch beträgt etwa 1 h, wobei die Dokumentation durch einen der Untersucher (Logopädie/Phoniatrie) erfolgt, während der andere Untersucher (Logopädie) die Beratung durchführt.

#### Follow-up

Kontrollen während und nach der R(C)T finden zu standardisierten Zeitpunkten statt (Tab. 1):

**Tab. 1:** Zeitpunkte und Inhalte der logopädischen Nachuntersuchungen bei Radio(chemo)therapie (R[C]T)

Zeitpunkt	Inhalt der Konsultation
–	*Logopädische Kontrolle/Prähabilitation*
*3. Woche der R(C)T („face-to-face“)* *5. Woche der R(C)T („face-to-face“)*	Zusätzliche Übungen oder Übungsanpassungen
	Zusätzliche Empfehlungen entsprechend der aktuellen Beschwerden
–	*Klinische logopädische Kontrolle*
*2 Wochen nach R(C)T („face-to-face“ oder telefonisch)* *6 Monate nach R(C)T (telefonisch)* *12 Monate nach R(C)T (telefonisch)*	Erfassung noch bestehender Beschwerden (z. B. Xerostomie, Lymphödem), Gewicht und Ernährungsstatus
	Abgabe von Empfehlungen bei persistierenden Beschwerden
	Einleitung weiterer Interventionen (z. B. lymphologische Therapie, Akupunktur)
*Bei Bedarf*	Zwischenzeitliche Therapien

#### Posttreatment Assessment

Ein Posttreatment Assessment findet 3 und 18 Monate nach der R(C)T statt, um kurz- und langfristige Beeinträchtigungen der Schluckfunktion, Stimmfunktion und/oder der Artikulation zu identifizieren und ggf. rechtzeitig intervenieren zu können. Hierbei werden alle Aspekte des Pretreatment Assessments wiederholt (Abb. 2).

Nach operativer Tumorresektion findet das Posttreatment Assessment 3 Monate postoperativ statt und erfolgen anschließend nur dann weitere Kontrollen, wenn noch relevante Beschwerden bestehen.

Bei geplanter adjuvanter Radiotherapie werden Patienten in das Prähabilitations-Schema aufgenommen und entsprechend betreut.

### Bisherige Erfahrungen

Insgesamt haben wir in der jetzigen Form des Zürcher Prähabilitationskonzepts im Zeitraum von August 2020 bis August 2025 bereits über 400 Patienten untersucht und behandelt. Dabei zeigt sich überwiegend eine hohe Compliance. Während zu Beginn unserer Sprechstunde v. a. organisatorische Schwierigkeiten (fehlende Zuweisungen, Planungsfehler) dafür sorgten, dass Termine nicht stattfanden, nehmen die Patienten die Termine und Kontrollen zum jetzigen Zeitpunkt größtenteils wahr.

Die Face-to-Face-Termine während der R(C)T wurden zu etwa 70 % wahrgenommen

**Abb. 3:**
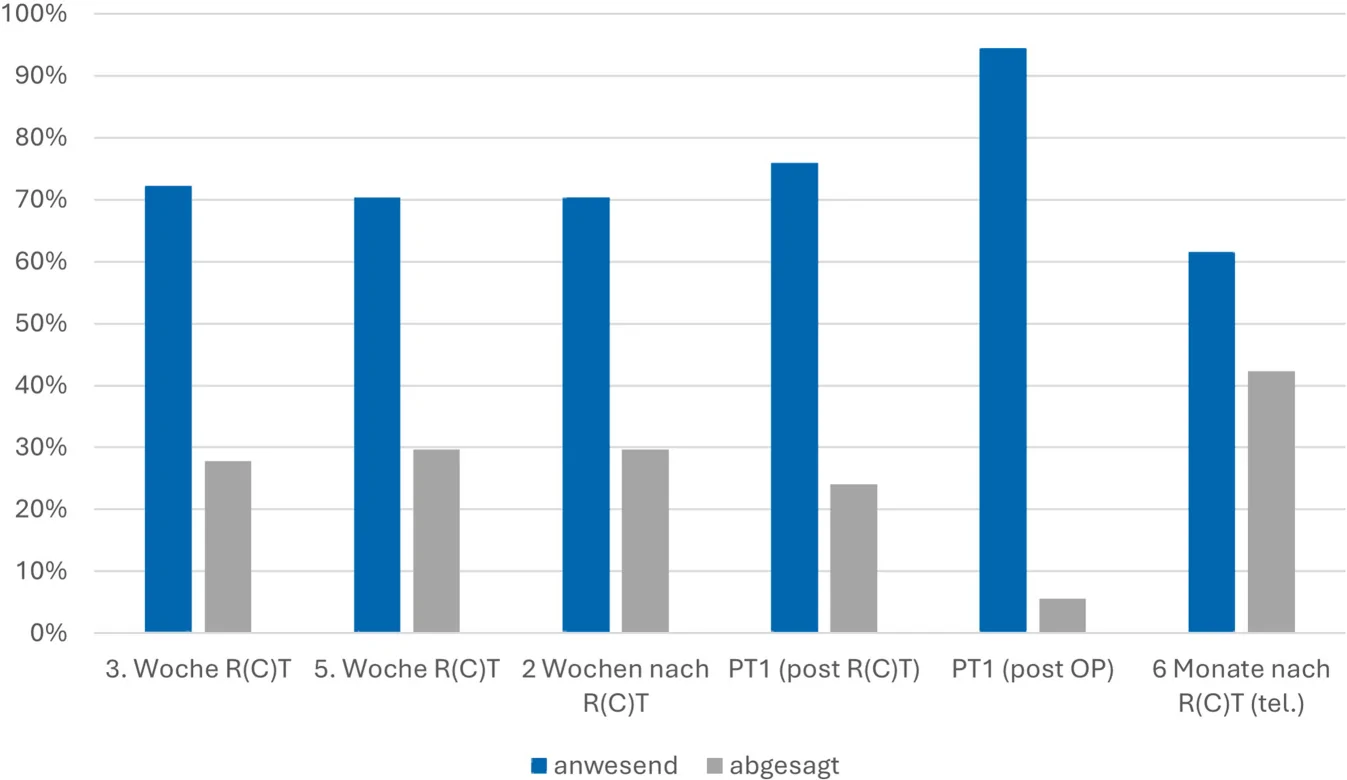
Prozentuale Anwesenheit vs. Terminabsagen/Abwesenheit (alle Absagegründe) pro Termin aller Patienten mit Pretreatment Assessment im Jahr 2024 und primärer Radio(chemo)therapie (R[C]T; *n* = 54) oder Op. (*n* = 18) (exklusive Patienten ohne allgemeine Einwilligung [Generalkonsent]); *PT1* Posttreatment Assessment 1 (3 Monate nach Behandlung)

Eine beispielhafte Analyse der Anwesenheiten vs. Terminabsagen aller von uns untersuchten Patienten mit Beginn einer primären R(C)T oder Op. im Jahr 2024 (Abb. 3) zeigt, dass die Face-to-Face-Termine während der R(C)T zu etwa 70 % wahrgenommen wurden. Posttreatment Assessments 3 Monate nach Behandlung wurden zu 76 % (post R[C]T) bzw. 94 % (post Op.) wahrgenommen. Das Posttreatment Assessment 18 Monate nach R(C)T ist in der vorliegenden Analyse noch nicht enthalten, da diese Termine größtenteils noch ausstehen.

**Abb. 4:**
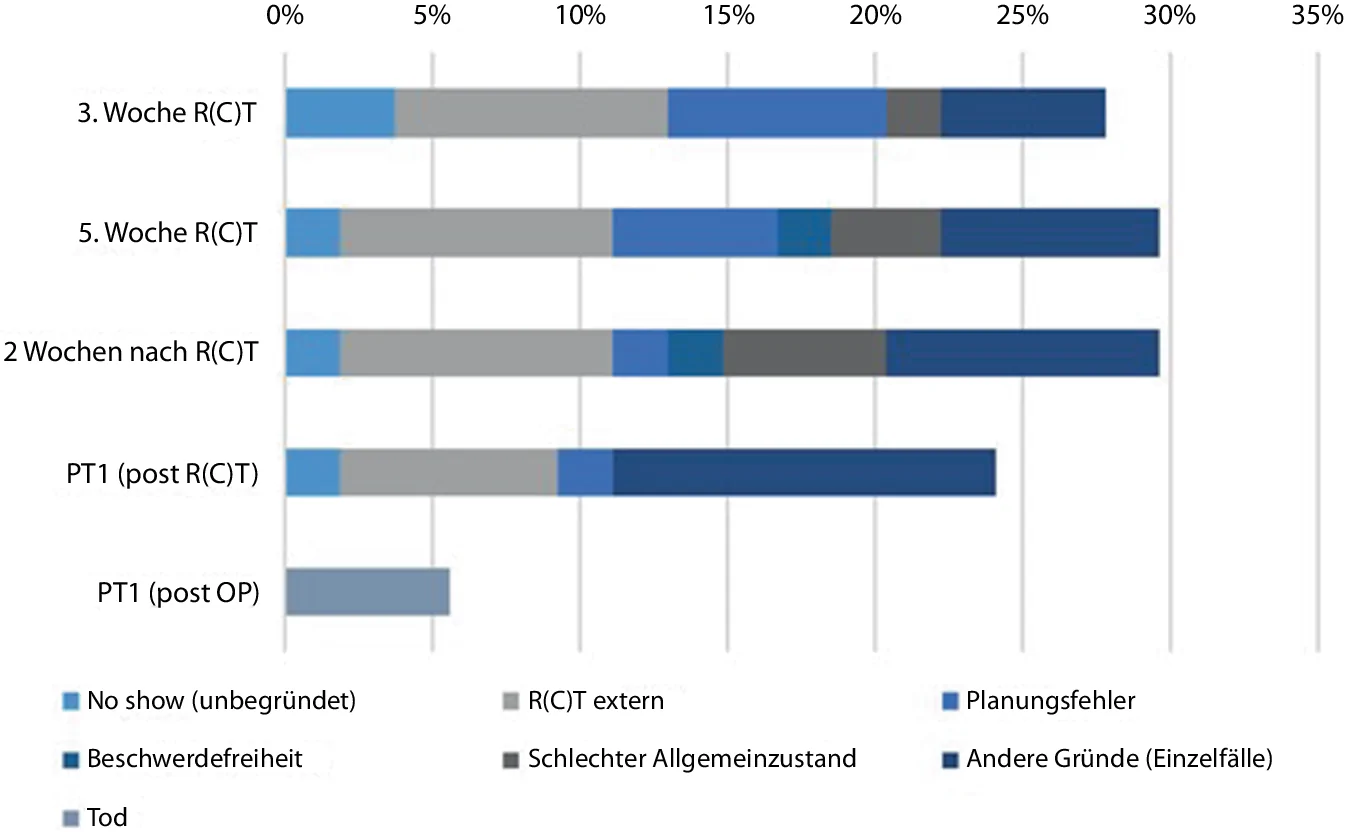
Gründe für Terminabsagen/Abwesenheit pro Termin aller Patienten mit primärer Radio(chemo)therapie (R[C]T; *n* = 54) oder Op. (*n* = 18) 2024 in % (exklusive Patienten ohne Generalkonsent)

Gründe für Absagen waren in der gleichen Analyse überwiegend die Durchführung der R(C)T in einer anderen Klinik sowie in Einzelfällen andere Gründe (z. B. längerfristige Auslandsaufenthalte, Abbruch der Behandlung; Abb. 4).

Da Patienten erfahrungsgemäß selbst bei Beschwerdefreiheit noch zu den Posttreatment Assessments 18 Monate nach der Behandlung erscheinen, ist zu vermuten, dass der Mehrwert der Konsultationen während der Termine für die Patienten deutlich wird. Dafür spricht auch das durchgehend positive verbale Feedback der Patienten bei Abschluss der Kontrollen, insbesondere bei Patienten, die eine R(C)T erhalten haben.

### Herausforderungen

Trotz der positiven Erfahrungen mit der Etablierung der Spezialsprechstunde bestehen verschiedene Herausforderungen in der praktischen Umsetzung:

Nach primären Operationen erfolgt die Information über eine anschließende adjuvante Radiotherapie nicht immer zeitgerecht, was die terminliche Planung der Sprechstunde erschwert.

Bei telefonischen Konsultationen können Patienten gelegentlich beim ersten Kontaktversuch nicht erreicht werden; mangels Kapazitäten für zusätzliche Anrufversuche entfällt der Termin in diesen Fällen. Dies spiegelt sich auch in der Analyse der Terminanwesenheiten 2024 wider: Hier konnten nur 62 % der bereits geplanten Konsultationen 6 Monate nach R(C)T durchgeführt werden.

Zum Zeitpunkt der Etablierung der Sprechstunde (2019) wurde der 100-ml-Wasserschlucktest eingesetzt. Die aktuelle Leitlinie empfiehlt inzwischen den 51-ml-Test. In unserer Sprechstunde wird das etablierte Verfahren zu Studienzwecken aktuell weiterhin genutzt, da die diagnostische Beurteilung der Schluckfunktion primär auf der FEES basiert. Eine Anpassung an die Leitlinie sollte geplant werden.

Der Betrieb der Sprechstunde erfordert erhebliche personelle Kapazitäten. Eine Auswertung der Konsultationen im Jahr 2024 zeigt die hierfür notwendigen Zeitressourcen auf (Tab. 2). Da bisher keine gesicherte Finanzierung besteht, gehen diese Kapazitäten teilweise zulasten anderer Bereiche.

**Tab. 2:** Personalaufwand in Stunden pro Monat 2024

Anzahl Konsultationen und Personalaufwand 2024		
Konsultationsart	Anzahl	h/Monat
*Pretreatment Assessment, (Tandemuntersuchung à 60* *min)*	108	18,0
*PT1 post Op. (3 Monate nach Op.), (Tandemuntersuchung à* *60* *min)*	22	3,7
*PT1 (3 Monate nach R(C)T), (Tandemuntersuchung à 60* *min)*	67	11,2
*PT2 (18 Monate nach R(C)T), (Tandemuntersuchung à 60* *min)*	36	6,0
*Patienten mit Start R(C)T, (→ 5× 30* *min Prähabilitation und Follow-up innerhalb 12 Monaten)*	84	17,5

*PT* Posttreatment Assessment, *R(C)T* Radio(chemo)therapie

## Fazit für die Praxis

- Ein instrumentelles logopädisches oder logopädisch-phoniatrisches Pretreatment Assessment vor Radio(chemo)therapie (R[C]T)/Op. ermöglicht potenziell eine patientenzentrierte Behandlung und erlaubt die Ableitung therapeutischer Maßnahmen bei bereits vor der Tumorbehandlung bestehenden Beschwerden.
- Unsere bisherigen Erfahrungen mit dem entwickelten Modell für Pretreatment Assessment und Prähabilitation bei Kopf-Hals-Tumoren lassen es sinnvoll erscheinen, eine logopädische Prähabilitation im Rahmen der Behandlung von Kopf-Hals-Tumoren (KHT) zu etablieren.
- Ziel der dabei durchgeführten und hier beschriebenen Maßnahmen ist es, ein gutes funktionelles Outcome der Schluckfunktion und eine verbesserte Lebensqualität langfristig zu gewährleisten.
- Das Posttreatment Assessment ermöglicht frühzeitige Intervention bei Spätfolgen oder persistierenden Beschwerden.
- Unsere Sprechstunde ermöglicht einen Beitrag zur Forschung bezüglich der in der Leitlinie „S3-Leitlinie Oropharyngeale Dysphagie infolge einer Kopf-Hals-Tumor-Erkrankung – Diagnostik und Therapie“ der Arbeitsgemeinschaft der Wissenschaftlichen Medizinischen Fachgesellschaften e. V. (AWMF) benannten Fragestellungen.
- Interdisziplinäre Zusammenarbeit ist für die erfolgreiche Umsetzung dieser Spezialsprechstunde unerlässlich.
- Der Betrieb der Spezialsprechstunde erfordert feste Personalressourcen und sollte entsprechend eingeplant werden.

## Data Availability

Die für diese Arbeit relevanten aggregierten Daten sind im Artikel sowie den zugehörigen Abbildungen und Tabellen enthalten. Darüber hinausgehende zugrunde liegende institutionsinterne Routinedaten sind aus datenschutzrechtlichen und institutionellen Gründen nicht öffentlich verfügbar.
